# A Case of Concomitant Lip Injury and Facial Pressure Ulcer in Lumbar Intradural Tumor Surgery With Repeated Transcranial Electrical Stimulations

**DOI:** 10.7759/cureus.31560

**Published:** 2022-11-16

**Authors:** Ryuta Matsuoka, Yasushi Shin, Rinsei Tei, Eiji Wada, Yasushi Motoyama

**Affiliations:** 1 Neurosurgery, Osaka Police Hospital, Osaka, JPN; 2 Spine and Spinal Cord Center, Osaka Police Hospital, Osaka, JPN

**Keywords:** motor evoked potential, transcranial electrical stimulations, lumbar intradural tumor surgery, pressure ulcer, lip injury

## Abstract

Transcranial motor evoked potential (MEP) is a common method in spinal surgery but requires strong electrical stimulation. Frequent transcranial stimulations can cause bite injury. In addition, a facial pressure ulcer is a problem in spinal surgery requiring prone positioning. We present a case of bite injury and facial pressure ulcer in prolonged lumbar tumor surgery with repeated transcranial stimulations. A 74-year-old woman developed left lower limb and low back pain. MRI revealed an intradural extramedullary tumor at L1. We performed tumor resection surgery. A silicon bite block was used, and the patient’s head was placed on a sponge headrest. The tumor was a schwannoma originating from the nerve root that innervated the left anal sphincter. Intracapsular resection was performed while referring to the frequent transcranial MEP monitoring. The left lower limb and low back pain improved after surgery; however, lip injury and facial skin ulcer occurred. The face showed marked swelling and was painful, so oral intake was difficult for a week. Wound healing was obtained three months postoperatively, but hypoesthesia remained. When using MEP in prolonged spine surgery with a headrest, it is necessary to pay attention to both bite injury and facial pressure ulcer. Intraoperative assessment of the face, number of transcranial stimulations, types of a bite block, and headrest may be important.

## Introduction

Transcranial motor evoked potential (MEP) is a widely used technique in spine surgery. Intense stimulation is required to transmit the stimulation to the brain beyond the skull. Therefore, electrical stimulations can induce body movements, which can cause bite injury. Bite injuries include lip, buccal mucosa, and tongue injuries [1]. The incidence of MEP-associated bite injury ranges from 0.1% to 6.5% [1-2]. A facial pressure ulcer is also a risk in spinal surgery requiring prone positioning [3]. The incidence of facial pressure ulcers after a prone spine surgery using a headrest (>3 h) is 27.3% [4]. In this article, we present a case of concomitant lip injury and facial pressure ulcers in prolonged lumbar surgery with transcranial MEP monitoring.

## Case presentation

The patient was a 74-year-old woman. She visited our hospital because of pain in her left lower limb and low back pain for five months.

The lower limb muscle strength was normal, and walking was stable. The patient felt pain in the lower back, posterior surface of the left thigh, and lower leg. Intermittent claudication was observed after walking for approximately 10 min. She has no bladder and rectal disorder.

**Figure 1:**
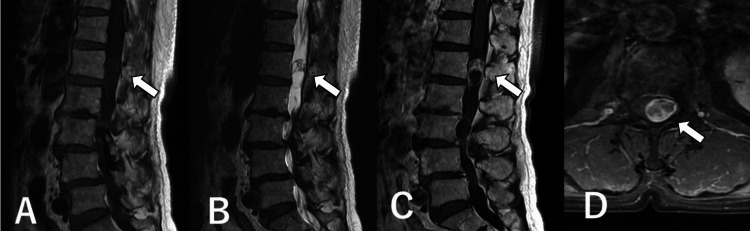
MRIs of lumbar tumor. A–C: Sagittal MRIs. An intradural extramedullary tumor (arrow) occupied the spinal canal and was low intense on the T1-weighted image (A) and high intense on the T2-weighted image (B). The contrast-enhanced T1-weighted image shows that a part of the tumor (arrow) consisted of cystic components, and the parenchymal component had a contrast effect (C). D: Axial fat-saturated contrast-enhanced T1-weighted image at L1. The tumor (arrow) compresses the spinal cord from the left side.

MRI revealed an intradural extramedullary tumor at L1. The tumor occupied the spinal canal and was a low intensity on the T1-weighted image and a high intensity on the T2-weighted image (Figure 1A,B). Part of it had cystic components, and the parenchymal component had a contrast effect (Figure 1C,D). The tumor was on the left side, compressing the conus medullaris and cauda equina. The radiological diagnosis for the tumor was a schwannoma. Since the tumor was the cause of low back pain and leg pain, we decided on surgical treatment.

**Figure 2:**
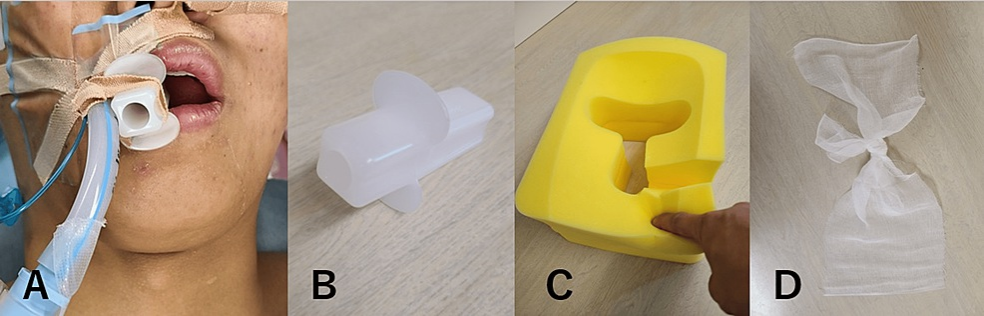
Pictures of used devices. A: The lower face of other patient in whom the same set up was used. B: A polyethylene bite block. C: A sponge headrest with a finger pressing on the soft surface. D: Two pieces of gauze tied together.

After the induction of general anesthesia, the intubation tube was fixed to the corner of the mouth, and a polyethylene bite block (MD-09011; Sumitomo Bakelite, Tokyo, Japan) was fixed in the middle with tape (Figure 2B). The intraoperative position was prone, and the patient’s head was placed on a sponge headrest (4088; Senshin Medical, Tokyo, Japan) (Figure 2C). Two pieces of gauze tied together were placed in the oral cavity (Figure 2D).

Transcranial MEP, triggered electromyography (EMG), and bulbocavernosus reflex (BCR) were prepared as intraoperative neuromonitoring. Corkscrew stimulation electrodes were placed on the C3/C4 position (international 10-20 system) for transcranial MEP. Surface stimulation electrodes were placed on the clitoris (cathode) and the adjacent labia (anode) for BCR. Needle recording electrodes were placed on the abductor pollicis brevis (APB), quadriceps, hamstrings, tibialis anterior (TA), hamstrings, gastrocnemius (GC), abductor hallucis (AH), and anal sphincter muscles. Neuromaster G1 (Nihon Kohden, Tokyo, Japan) was used as a neurophysiologic monitoring system.

We performed L1-2 left hemilaminectomy and dural incision. A tumor originating from the cauda equina was confirmed. Tumor resection was performed under microscopy. Intraoperative frozen pathological examination confirmed schwannoma. The triggered EMG revealed that the nerve in which the schwannoma developed innervated the left anal sphincter. Therefore, complete dissection of the tumorous nerve root was avoided, and intracapsular resection was performed.

The intensity of the transcranial MEP was set to 130 mA, and a dedicated monitoring technician applied the stimulation. Five train biphasic stimulations were applied. Transcranial stimulation was performed at least once within 10 min, and more frequent stimulation was applied during procedures with a high risk of nerve damage. BCR monitoring was done at the same timing as MEP.

**Figure 3:**
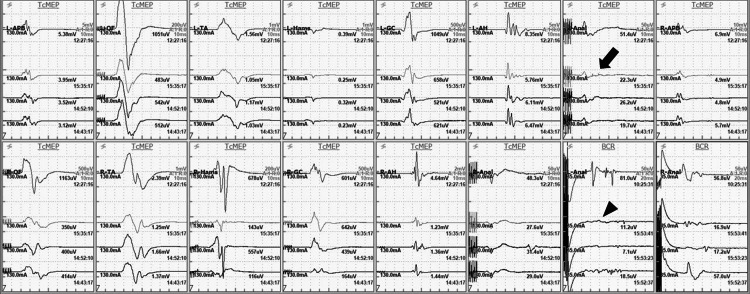
Waveforms of transcranial motor evoked potential and bulbocavernosus reflex monitoring. The APB, QF, TA, Hams, GC, AH, and Anal were the recording muscles. The waveform located at the top of each frame was the baseline. The three lower waveforms were at the end point of tumor resection. The amplitudes of the left anal sphincter (arrow) and left BCR (arrow head) decreased by >50% compared with the baseline waveforms. APB, abductor pollicis brevis; QF, quadriceps femoris; TA, tibialis anterior; Hams, hamstrings; GC, gastrocnemius; AH, abductor hallucis; Anal, anal sphincter

Near-total resection was achieved and the amplitudes of the left anal sphincter and left BCR were reduced by ≥50% compared with the baseline waveforms, so the resection procedure was finished (Figure 3). The operation time was 6 h.

**Figure 4:**
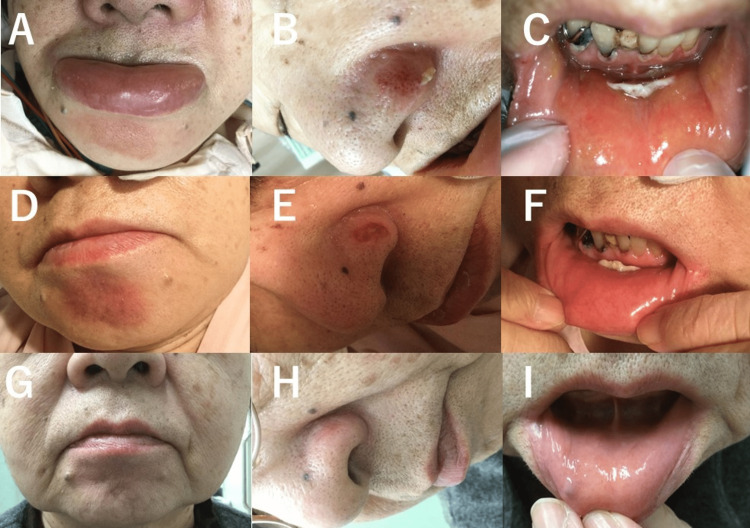
Postoperative photos of the face. A–C: Photos of the day after surgery. A: Facial pressure ulcer of the chin and the marked swelling lip and chin. B: Facial pressure ulcer of the left nose. C: Laceration on the inside of the lower lip. D–F: Photos one week after surgery. D: Epithelialization of the facial pressure ulcer of the chin. Mild facial swelling. E: Epithelialization of the facial pressure ulcer of the left nose. F: The lip injury remains. G–I: Photos three months after surgery. G, H: Healed facial pressure ulcers of the chin and left nose. I: healed lip injury.

The left lower limb and low back pain improved after surgery. However, abrasion of the chin and left nose occurred (Figure 4A,B). They were facial pressure ulcers, and a laceration on the inside of the lower lip was also observed (Figure 4C). This wound was created by being sandwiched between the bite block and front teeth. On the day after the surgery, there was pain and marked swelling of the face, mainly in the chin and lip. Oral intake was difficult, so an intravenous drip was needed for about one week. Moreover, 0.033% dimethyl isopropylazulene ointment was used for the treatment of facial pressure ulcer. A mouthwash was prescribed for lip injury.

At one week after surgery, epithelialization of the facial pressure ulcer progressed, and the ointment was changed to 0.003% alprostadil alfadex. Although the lip injury had not healed yet, the swelling of the chin and lip had reduced (Figure 4D,F). She complained of hypoesthesia in the chin and started taking mecobalamin 1.5 mg/day.

At three months after the surgery, the facial pressure ulcer and lip injury had healed, and the ointment treatment was completed (Figure 4G,I). The administration of mecobalamin was also terminated, but mild hypoesthesia of the chin remained.

Informed consent was obtained from the patient for the publication of this case report and any accompanying images.

## Discussion

This report emphasizes an important point. In lumbar surgery with MEP, which often uses a headrest, it is necessary to pay attention to the occurrence of both bite injury and facial pressure ulcer. Especially, in spinal tumor surgery, bite injury and facial pressure ulcers are likely to occur because the operation time tends to be long and transcranial stimulation is frequently applied to check motor function. In the case of prolonged surgery, it is necessary to pause the procedure at regular intervals to decompress the face and check the wound [5].

The incidence of bite injury varies among reports. In one retrospective study where oral surgeons evaluated bite injuries, 12 of 186 patients (6.5%) had bite injuries [1]. Of these, lip injuries occurred in 3 (1.6%) patients [1]. Bite injury occurs at a frequency that can never be ignored.

In this case, the cause of severe lip injury was repeated transcranial stimulations over a long period. Essentially, unnecessary transcranial stimulations should be avoided, but in this case, the triggered EMG of the tumoral root induced the reaction of the anal sphincter, and frequent MEP stimulations were required. Sacrificing the tumoral root from which a positive myogenic response can be evoked may result in motor deterioration [6]. Therefore, we performed tumor resection while confirming MEP response.

The C3/4 stimulation might produce stronger biting than C1/2 stimulation because the electrodes are closer to the facial motor cortex, jaw muscles, and trigeminal nerves [7]. However, since the electric field of C1/2 stimulation becomes shallower [8], a stronger stimulation is required by inducing MEP of the lower limbs compared with C3/4 stimulation, body movement may become larger in C1/2 stimulation.

Triggered EMG can evoke muscle response even with very low stimulation intensity. No stimulation is required for monitoring free-run EMG. The triggered and free-run EMG technique is useful for treating spinal schwannoma [9]. Reducing the frequency of transcranial stimulation and increasing the frequency of using triggered and free-run EMG monitoring are ways to reduce body movements. In S2-S4 roots, BCR monitoring can also be implemented.

A bite block is indispensable for the prevention of bite injury. In our case, we used a hard bite block made of polyethylene, but it caused a marked lip injury. A hard bite block may accumulate the occlusal force and possibly promote tooth injury and laceration of the tongue and lip [10]. A soft bite block with rolled-up gauze should be an effective precautionary measure for bite injury [10]. The bite block should be large enough to prevent the teeth from becoming close to the tongue and is secured properly to minimize the chance of shifting [2]. And the bite blocks should be also kept between the molar teeth on both sides, keeping only one in the middle is a risk [5]. Furthermore, a mouthpiece is also considered effective in preventing bite injury [11]. 

On the contrary, a facial pressure ulcer is an unavoidable complication. In brain surgery and cervical spine surgery, even if the patient is in the prone position, the head is fixed using a Mayfield head clamp, so the face is not in contact with anything. In lumbar spine surgery, a facial pressure ulcer is a problem because a headrest is often used.

In the present case, we used a sponge headrest, which resulted in a facial pressure ulcer. The pressures measured for the protected helmet system were lower for both the forehead and chin in comparison with the sponge headrest [3]. Studies have also reported reducing facial pressure ulcers such as using the Mayfield cramp to prevent facial pressure ulcers in sacral tumor resection and facial protection with paraffin tulle gras dressing to cover bony prominences [12-13].

Although the risk factors for developing facial pressures ulcer include hypotension, higher temperature, prolonged operation time, and much crystalloid therapy, a study reported that facial movement due to repeated neuromonitoring stimulation during long procedures causes shear forces on the face leading to pressure ulcers [4, 13]. So far, facial pressure ulcer has rarely been classified as an MEP-related complication, but facial pressure ulcer is possibly related to friction and change in the position of the face because of body movements by repeated transcranial stimulations. In this case, the lower lip was pushed in by a headrest touching the chin and repeated transcranial stimulations. Consequently, the lip was sandwiched between the bite block and the front teeth. This phenomenon may confirm that MEP is causing the shear force between the headrest and the chin.

In our opinion, there were two main causes of lip injury. One was the jaw movement by repeated electrical stimulations and insufficient bite block, and the other was the soft sponge headrest causing the poor positioning of the patient face. The combination of them caused slipping the lip between the hard bite block and the front teeth.

## Conclusions

The bite injury and facial pressure ulcer are related complications when using MEP in lumbar surgery with a headrest. The combination of these two complications on the face further reduces the postoperative satisfaction of patients. Frequent intraoperative checking of the face, using triggered and free-run EMG to reduce the number of transcranial stimulations, large soft bite blocks not only between the incisors but also between the molar teeth, and using Mayfield cramp or protected helmet system may be recommended.
